# An *a posteriori* measure of network modularity

**DOI:** 10.12688/f1000research.2-130.v3

**Published:** 2013-12-27

**Authors:** Timothée Poisot

**Affiliations:** 1 Département de Biologie, Chimie et Géographie, Université du Québec à Rimouski, Rimouski, G5L 3A1, Canada; 2 Québec Centre for Biodiversity Science, Montréal, H3A 1B1, Canada

**Keywords:** nodes, network, modularity, interactions

## Abstract

Measuring the modularity of networks, and how it deviates from random expectations, important to understand their structure and emerging properties. Several measures exist to assess modularity, which when applied to the same network, can return both different modularity values (i.e. different estimates of how modular the network is) and different module compositions (i.e. different groups of species forming said modules). More importantly, as each optimization method uses a different optimization criterion, there is a need to have an a posteriori measure serving as an equivalent of a goodness-of-fit. In this article, I propose such a measure of modularity, which is simply defined as the ratio of interactions established between members of the same modules vs. members of different modules. I apply this measure to a large dataset of 290 ecological networks representing host–parasite (bipartite) and predator–prey (unipartite) interactions, to show how the results are easy to interpret and present especially to a broad audience not familiar with modularity analyses, but still can reveal new features about modularity and the ways to measure it.

## Introduction

Modularity, the fact that groups of nodes within a network interact more frequently with themselves than with other nodes, is an important property of several systems, including genetic ^1, 2^, informatics ^3^, ecological ^4^, and socio-economic ^5^ interactions, as well as biogeographic patterns ^6, 7^ and disease spread management ^8^. Because of the relevance of modularity for network properties, it is important to assess it correctly. Several methods exist to measure network modularity, some of which rely on the optimization of a given criterion ^9, 10^, label propagation ^11^, or combination of these approaches ^12, 13^. These methods return two elements. The first is a value of modularity for the networks, most often within the 0–1 interval. Each method often has a threshold value, above which a network is considered to be modular. Increasing values reflect an increasingly modular structure. The second element is a "community partition", *i.e.* the attribution of each node to a module.

Recently, Thébault ^7^ showed that different measures of modularity tailored to presence/absence matrices ( *i.e.* networks in which links have no weight), gave roughly equal estimates of the significance of modularity, but differed in the community partition they returned ( *i.e.* the identity of nodes composing each module varied). In such situations, one might look for a way to choose which community partition should be used. The challenge in this situation is that the criteria used by each optimisation method cannot be meaningfully compared, and so there is a need for *a posteriori *measurement of how strong the modular structure is, regardless of the method used to obtain the community partition. More importantly, this criterion should be different than the one used to track the progress of any optimisation algorithm.

An important feature of modular networks is the occurrence of interactions between nodes of different modules. They contribute to the propagation of disturbances ^4^, flow of information ^14, 15^, and cross-regulation of biological processes ^16^, *inter alia* ^17^. In addition to measuring how modular the network is, determining to what extent modules are connected, and to identify nodes and edges responsible for connecting modules, is thus valuable information. In this article, I propose an *a posteriori* measure of the proportion of interactions established between modules, *i.e.* edges connecting different communities. I apply this measure to the community partition identified by the Louvain method on 290 ecological networks, and show that it behaves in a similar way to other modularity measures.

## The measure

In this contribution I define the *realized modularity*, termed *Q _R_*. *Q _R_* measures the extent to which edges, within a network, are established between nodes belonging to the same module. For *E* edges in a network, if *W* of them are established between members of the same module, then

$$Q_{R}\;=\frac{W}{E}. (1)$$

When there are no between-module links, then *W* = *E* and *Q _R_* takes the maximal value of 1. When between-module interactions are as numerous as within-module interactions, then *W* = *E*/2, and *Q _R_* takes the minimal value of 1/2. To express the *realized modularity* as a value between 0 and 1, it is expressed as:

$${Q^{\prime }}_{R}=\;2\;\times Q_{R}-\;1. (2)$$

**Figure 1.**
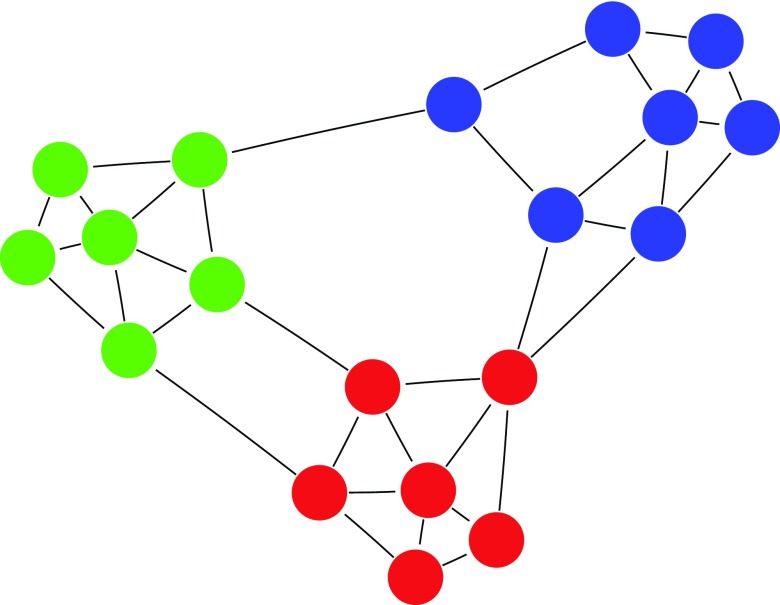
A cartoon depiction of a modular network with links between modules. Nodes of the same modules are identified by different colors. This network has a modularity (Louvain method) of *Q* = 0.527. Out of the 36 interactions, 31 are established within modules, and 5 between modules. This gives a *Q _R_* value of 0.86, and *Q′ _R_* = 0.72.

Note that *Q′ _R_* will yield values in the [0; 1] interval only if there are more edges established within than between modules. Although, if modules are determined at random, *Q′ _R_* values are expected to be centered on 0, it is expected that they will increase when modules are properly optimized (only as far as the network is modular). The main advantage of *Q _R_* is that it is agnostic with regard to the measure used to optimize modularity (and even to the method by which the nodes were assigned to modules, which can be arbitrary), as it acts *a posteriori*, *i.e.* after nodes have been attributed to modules. Nonetheless, it assumes a simple yet functional definition of modularity: the fact that nodes interact more within than between modules. Given that measuring to which extent this is true, it can therefore be used to select the community detection method maximizing modularity. This measure works on most types of networks, as it makes no difference if links are directional, or if the networks are bipartite/unipartite. An illustration of this measure is given in Figure 1. This measure is purposefully simple, (i) so that it makes only minimal assumptions about what modularity is (except for the fact that in a modular network, nodes interact more within than between modules), or how it should be optimized, and (ii) because it is not meant to be used to optimize modularity, but to either compare the outcome of different methods, or present the value of modularity in a way that is straightforward to interpretate.

A python implementation of this measure, using the networkx package, is proposed at https://gist.github.com/tpoisot/4947006. It reads data in the edge list format, and offers additional functions to generate null networks, as detailed in the following section.

## Example application: realized modularity in ecological networks

In this section, I analyze the modular structure of a large dataset of 290 ecological networks (187 food webs and 113 host-parasite networks) published in previous meta-analyses ^18, 19^. Modularity is an important feature of ecological interaction networks, which is linked to their resilience ^20, 21^, stability ^7^, biogeographic structure ^22^, functioning ^23^, and to the evolutionary mechanisms involved in their assembly ^24^. Notably, the occurrence of interactions between and within modules plays a central role in the structure of pollination networks ^4^, and help buffer the effect of species extinctions ^21^.

The existence of interactions in ecological systems involves a large family of processes, ranging from abudance related ^25, 26^ (abundant species are more likely to interact together) to trait related ^27^ (pollination depends on the flower and insect having compatible morphologies, predators are constrained by the body-size of their preys). The interaction within these different families of mechanisms will drive heterogeneity in interaction strength ^28^. Yet, the analysis of binary matrices (is there an interaction between a pair of species, or not), still has relevance to identify properties that are conserved across systems ^29^, especially given that one could argue that quantitative information on interaction strength is an additional level of information. The systems analyzed in this section are represented by their adjacency matrix, describing the presence or absence of an interaction. Bipartite networks have further been transformed into unipartite networks before analysis.

### Data and analysis

I used the Louvain method ^30^ to detect modules, due to its rapidity and efficiency on large networks. The Louvain method works in two steps: first it optimizes modularity *locally*, through clustering of neighboring nodes. These clusters are, in the second steps, aggregated together, until modularity ceases to increase. This method is known to give values of modularity comparable to what is found using *e.g.* simulated annealing, and has been observed to give modules that have a functional relevance ^30^. Once the partition is returned by the Louvain method, I recorded its realized modularity *Q′ _R_*, and its modularity *Q* (using the Newman and Girvan ^31^ measure).

For each network, I compared the values of *Q* and *Q′ _R_* on the empirical networks to their random estimate using a network null model. Because random networks will by chance (here meaning, as expected by networks having a given connectance and thus degree distribution, Poisot and Gravel ^32^) display a modular (among other) structure, it is important to confront the empirical measures of both *Q* and *Q′ _R_* to their random expectations. The null model is defined as follows. For each node *n* of the network, I measured its degree *d _n_*, its number of successors (the number of node it links to, or generality in ecological terms, as *per* ^33^) *g _n_*, and its number of predecessors (the number of nodes that link to it, or vulnerability) *v _n_.* In each random network, for each pair of nodes ( *i*, *j*), the probability that *i* interacts with *j* is given by

$$P(i\;\to \;j)\;=\;\frac{1}{2}\;\left(\frac{g_{i}}{d_{i}}\;+\;\frac{v_{j}}{d_{j}}\right), (3)$$

and conversely for *P*( *j* → *i*). This null model allowed the generation of pseudo-random networks through a Bernoulli process (in each replicate, the occurrence of a link is randomly determined), with the same expected connectance, and the same expected distribution of degrees, generality, and vulnerability, as the original one (these properties are also conserved at the *node* level). For each of the 290 networks, 1000 pseudo-random replicates are generated. For each of them, the average value of *Q _R_* and *Q′ _R_* are estimated along with their 90% confidence interval. When the empirical value lies outside the confidence interval, it can be assumed that the modular structure of the network is different than expected by chance.

### Results and discussion

**Figure 2.**
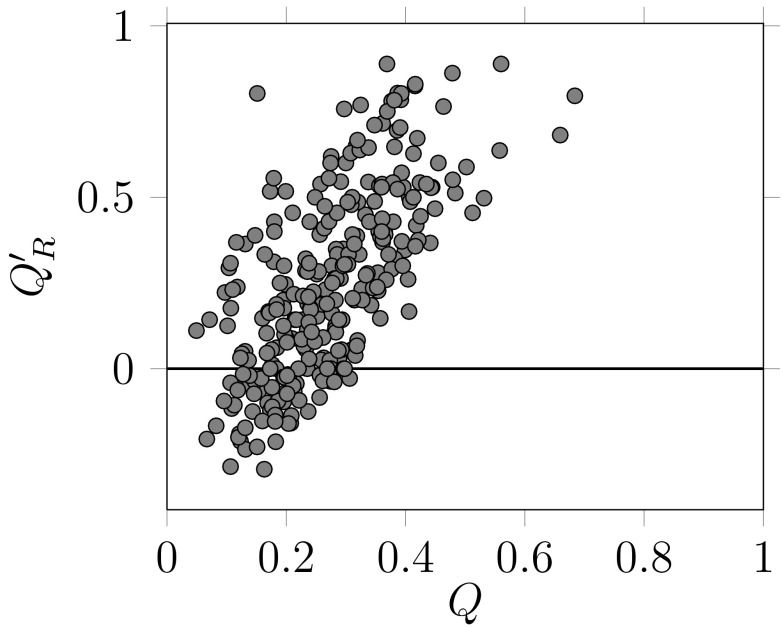
Relationship between the modularity of the best partition using the Louvain method and the *a posteriori* realized modularity. There exists a strong, positive relationship between the two variables. Worth noting is the fact that, for some networks, the best partition resulted in *negative* versions of *Q′ _R_*, *i.e.* there were more interactions between than within modules. Each dot corresponds to a network.

**Figure 3.**
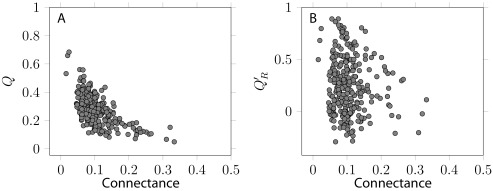
Relationship between the two measures of modularity and network connectance. **A**. *Q* is negatively affected by connectance, *i.e.* densely connected networks are more likely not to be modular. **B**. *Q′ _R_* is not affected by connectance, allowing to use it to compare different networks. Each dot corresponds to a network.

There is a strong, positive relationship, between the values of *Q′ _R_* and the values of *Q* (Pearsons's product-moment correlation coefficient, as implemented in R 2.15 ^34^, *ρ =* 0.64, 288 d.f., *p* < 10 ^–6^), *i.e.* networks for which a high modularity is detected tend to have relatively few between-module links ( Figure 2). It is worth noting that some *Q′ _R_* values were negative: in some cases, the best community division resulted in more interactions between than within modules. This result highlights why using an *a posteriori* measure is useful: other measures of modularity do not reveal the fact that there were more interactions between than within modules. In the dataset examined, most of the networks with a modularity lower than 0.2 had a negative realized modularity. This result suggests that discussing the modularity of such networks makes little sense, as their modules are not more densely connected, within a module, than other random collections of nodes within the graph. *Q* and *Q′ _R_* have different relationships with connectance ( Figure 3). Increased connectance values resulted in lower modularity ( *ρ* = –0.61, 288 d.f., *p* < 10 ^–6^), but had no impact on *Q′ _R_*. This is a desirable property, as it allows easy comparison with the *Q′ _R_* values of networks with extremely different connectances.

**Figure 4.**
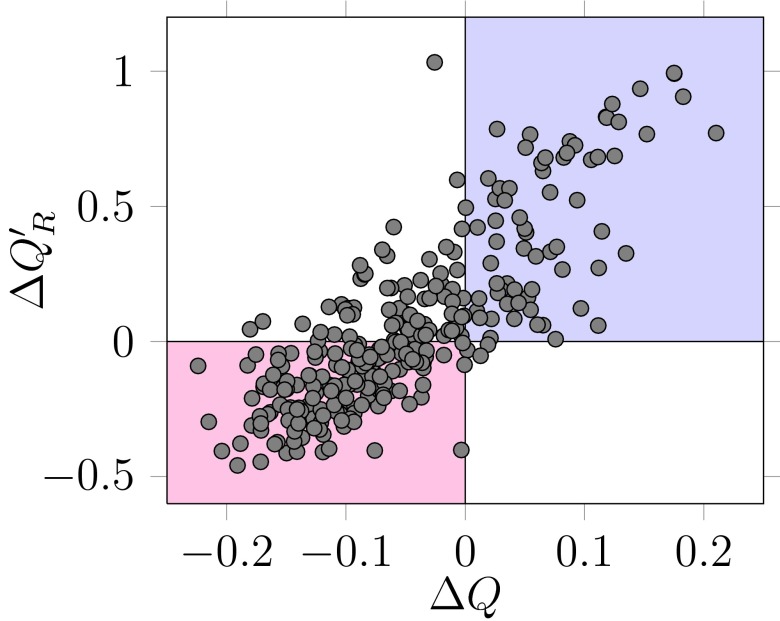
Linear relationship between the deviation from random expectation in *Q* and *Q′ _R_*. Networks in the red area are detected as being less modular than expected both by *Q′ _R_* and *Q*, while networks in the blue area are detected as being more modular. Although the agreement between the two measures is good (see main text for statistics), some networks are detected as having a higher than expected realized modularity *Q′ _R_*, despite a lower than expected modularity *Q*. Each dot correspond to a network.

There is a linear relationship between the deviation from random expectation of *Q* and *Q′ _R_* ( *ρ* = 0.78, 288 d.f., *p* < 10 ^–6^ – Figure 4). The deviations (respectively Δ *Q* and Δ *Q′ _R_*) are calculated as the empirical value, minus the average of the values on the networks generated by the null model. As an example, a Δ *Q* less than zero indicates that the empirical network is less modular than expected by chance. Confidence intervals for the average of the null models were typically very narrow (not represented in the figure to avoid cluttering – see associated original dataset), probably owing to the fact that the null model is restrictive on the type of networks which are generated. It is worth noting that for some networks, the diagnostic of the null model analysis is conflicted. In a vast majority of the situations, this corresponds to networks having a lower modularity than expected by chance, yet having a higher realized modularity (dots in the upper left corner of Figure 4). In this type of situation, whereas one would usually conclude that the networks are not significantly modular, the identified modules are nonetheless more densely connected (internally) than they are with the rest of the network. Because the dataset presents these contrasted situations, it allows us to understand how the measure reacts to different network structures. Depending on whether the true modularity, or the realized modularity, is the most relevant metric of the processes studied, the interpretation of the null models for these networks will be different.

Relationships between raw and realized modularity Relationships between raw and realized modularity for 290 networks, including the results of null models
results.dat
w - web number
q - raw (Louvain) modularity
nm - number of modules
qr - realized modularity
ed - number of edges
no - number of nodes
co - connectance
qe - random expectation of Louvain modularity
eqe - variance of the random modularity expectation
qre - random expectation of realised modularity
eqre - variance of the random realized modularity expectation
rq - rank (based on modularity)
rqr - ranked (based on realized modularity)
dq - empirical - random modularity
dqr - empirical - random realized modularity
altmeasures.dat
w - network (unipartite) number
Wa(R) - modularity and realized modularity with the walktrap method
Sp(R) - with the spinglass algorithm
Eb(R) - with the edge-betweenness method

Finally, for the unipartite network dataset, I compare the results of three alternative methods of community detection (the walktrap, spinglass, and edge-betweenness methods, as implemented in the *igraph* library). For each of the unipartite networks, I computed the value of Barber's *Q*, and *Q′ _R_*, on the best partition found. The strong correlation between *Q* and *Q′ _R_* were observed for the spinglass method ( *ρ* = 0.61, 165 d.f., t = 10.02), and the weakest for the edge-betweenness method ( *ρ* = 0.04, non-significant at *α* = 0.05). The walktrap algorithm gave results in between ( *ρ* = 0.489, 165 d.f., t = 7.20). For both the walktrap and edge-betweenness methods, several networks had negative values of *Q′ _R_*, which indicates that the "best" community partition had more links between than within modules. The spinglass method had, by contrast, less than 8% of all networks with values of *Q′ _R_* lower than 0, meaning that this algorithm should be prefered when one wants to group nodes in densely connected clusters. This result reinforces the statement made by Thébault ^7^, i.e. that several modularity optimisation methods will return best modular structures that widely differ in their properties; thus, there is a need for *a posteriori* comparison of these outputs.

## Conclusions

The *Q′ _R_* measure presented here allows the estimation of the proportion of interactions established between different modules in a network. This measure can be analyzed much in the same way as other measures of modularity, but is applied *a posteriori.* As such, it can help choose the "best" community partition according to the property of the network that one wants to maximize. For example, choosing the partition giving the lowest *Q′ _R_* can help identify which species are more likely to act as connectors between different modules. Ultimately, this information may have some practical relevance as a decision tool. Saavedra *et al.* ^5^ showed that different nodes contribute differently to overall network properties. In a context in which networks are increasingly being used as management tools to adress *e.g.* conservation or pest management ^8^, knowing the realized modularity, and developing methods to estimate which species have the highest impact on it, can allow the design of efficient policies to maximize, or decrease, the ability of network modules to interact.
