# Supplementary material for: An a posteriori measure of network modularity
Source: F1000Res. 2013 Dec 27;2:130. Originally published 2013 May 23. [Version 3] doi: 10.12688/f1000research.2-130.v3 (PMC3901540; doi:10.12688/f1000research.2-130.v3)
Supplement: Peer review history

# Peer review: Referee response for version 3

**DOI:** 10.5256/f1000research.3306.r2914

**Published:** 2014-04-08

**Authors:** Daniel Carstensen

**Affiliations:** 1 Department of Bioscience, Universidade Federal de São Paulo, São Paulo, Brazil

I have no further issues with this submission.

I have read this submission. I believe that I have an appropriate level of expertise to confirm that it is of an acceptable scientific standard.

# Peer review: Referee response for version 3

**DOI:** 10.5256/f1000research.3306.r2912

**Published:** 2014-01-06

**Authors:** Jochen Fründ

**Affiliations:** 1 Agroecology, Georg-August-University of Göttingen, Göttingen, Germany

I appreciate the improvements to the article done during the revision. Both the abstract and the main text now describe better where the article is going and explain the purpose of the new metric. Several minor concerns have been overcome and questionable points have been clarified.

However, my main reservations largely persist. This includes:

1. I question whether Q' _R_ is generally useful for choosing which community partition to use. For choosing the best partition for a particular purpose, I think it would be most efficient to use the metric of choice during the optimisation and not *a posteriori*. In my view, a simple *a posteriori* metric is mostly useful for presenting and describing modularity. For this purpose, I would prefer Q _R_ over Q' _R_ because it makes even less assumptions.
2. Fig. 4 still suggests to me that most of the empirical networks used here are not more modular than expected by chance (more negative than positive deltaQ values) and I am thus still uncertain how suitable the dataset is for exploring modularity.
3. Less fundamentally, in the comparison of the different algorithms from the *igraph *package I am still missing the percentage of Q' _R_ that were below 0 for the walktrap and edge-betweenness method, in order to compare it to the 8% for the spinglass method (currently it only says "several").

In conclusion, I think the metric Q _R_ presented here can be helpful, but should be applied with caution.

I have read this submission. I believe that I have an appropriate level of expertise to confirm that it is of an acceptable scientific standard, however I have significant reservations, as outlined above.

# Response

**Published:** 2014-01-06

**Authors:** Timothée Poisot

**Affiliations:** Université du Québec à Rimouski, Canada

I believe these points were all addressed in the revision and associated replies. I would be willing to provide more arguments, but the referee is merely stating their feeling or ideas, not making any factual criticism of the paper. In these circumstances, it is very hard for me to decide what to revise.

# Peer review: Referee response for version 2

**DOI:** 10.5256/f1000research.2230.r2678

**Published:** 2013-12-05

**Authors:** Jochen Fründ

**Affiliations:** 1 Agroecology, Georg-August-University of Göttingen, Göttingen, Germany

This article suggests a simple intuitive measure of network modularity. The suggested measure, Q'R, is related to established measures but calculated slightly differently. It is proposed as an *a posteriori* measure, which means it is not suggested to be used for assigning nodes (e.g. species) to modules, but only to evaluate partitions based on other methods that calculate modularity, identify modules and assign nodes to them.

In principle I welcome the suggestion of a simple, easy to interpret measure. The metric suggested here can help presenting modularity. I see that the amount of between-module links in relation to within-module links may have direct functional consequences. Established measures of modularity measure basically the same, they only correct for the expectation of within-module links in non-modular random networks in a different way.

However, I have a number of serious concerns making the study potentially misleading. These concerns include reservations about whether the analyses address the study aim, whether the dataset is suitable for testing modularity, how the proposed metric is interpreted and that it is suggested as an *a posteriori *measure.

**General issues:**

The study aim set out in the abstract and introduction, to compare different methods and approaches detecting modularity, is not reflected by the analyses. Neither is a functional meaning of the new metric demonstrated to support the case that the metric can be used to evaluate other methods, and decide which method to use. The abstract claims that new insights are gained about the modularity of the food webs in the empirical example dataset, but I struggle to find these new insights.

A paragraph added during the revision does some comparison, but it is not integrated with the rest of the paper and neither does it demonstrate the usefulness or added value of the new measure. For the most part, the paper rather compares values of one standard measure of modularity with values for the new metric in an example dataset of 290 unipartite and bipartite food webs. Using one method of module assignment, the paper shows how the two metrics are related to each other, to randomizations of the webs, and to network connectance. The meaning of these relationships for the study purpose is unclear.

Importantly, the usefulness of the empirical dataset for evaluating modularity methods is questionable. Typically, studies proposing modularity methods test them on networks of known modularity. However, the nullmodel analysis brings to attention that the vast majority of the networks used here are not more modular (based on Q) than expected by chance, and even less might be significantly modular. This means that this study tries to evaluate modularity methods on networks that are mostly not modular. This questions the value of the whole study and calls again for external information for validation. If networks are not modular, then the practical value of measuring modularity becomes negligible: the variation in module assignment in networks not significantly modular is probably much less worrying than failure to detect a known modular structure (which is not given here).

The straightforward interpretation of QR is changed in Q'R, the version the author describes as being scaled between 0 and 1, only to report negative values later on in the paper (Fig. 2). For networks of unknown modularity, Q'R can actually take values between -1 and 1. Furthermore, the notion that negative values of Q'R detect cases of spuriously significant modularity is not generally correct. The threshold of meaningful modularity depends on the purpose and may be above or below Q'R =0.

This brings me to a fundamental problem with the study – what is modularity and why should it be measured? The author states that the new metric “makes no assumption about what modularity is”. If this is really the case, then there is no point in defining a measure for it. To be useful, an assumption about what is being measured has to be made. This questions the claims and even policy recommendations made by the author. The difference in concepts and goals is likely a major reason why previous methods differ (e.g. unipartite vs. bipartite modularity suggested by Guimera *et al.*, 2007). Only when a concept of interest is defined can methods be compared in how well they serve the purpose.

I am not sure how useful the whole idea of an *a posteriori *measure is. The author stresses that the measure is not aimed at maximizing modularity in an algorithm, but just to select which algorithm to use. This is not convincing: either the measure reflects the property of interest, then it should be maximized in the first place in the algorithm to find the best partition; or it is not a sensible measure, then it cannot be used for selection at all. The approach proposed here appears very inefficient and almost certainly not to give the best partition. Furthermore, any measure of modularity could be calculated *a posteriori* or during modularity optimization. The description of this index specifically as an *a posteriori* measure gives no real sense, without additional data or simulations showing that it is more meaningful than others. If the functional meaning was demonstrated, there could be some value in using it *a posteriori *for those who don’t have access to source algorithms.

**Alternative methods (algorithms) paragraph:**

As said above, this is not connected to the rest of the paper. Of course it improves the paper to consider alternative methods for module assignment. However, this paragraph has several shortcomings. First, restricting this analysis to the unipartite networks makes it hard to compare to the other results. Second, it remains unclear why this focuses on the correlation between Q and Q'R. The modularity of the partition returned by each method would be compared more directly by comparing the values of Q or Q'R between methods. At the moment, for judging the three methods the reader is just left at guessing that “several” negative values (for methods walktrap and edge-betweenness) are more than “less than 8%” (method spinglass). Third, it looks like an inconsistent ad-hoc addition: citations for the methods and the igraph library (package) are missing, the methods are not mentioned before or described and correlation coefficients are called r here but rho above.

**Null model:**

The description of the nullmodel leaves unclear whether the connectance and degree are fixed exactly or just determine the expected value probabilistically. Moreover, the nullmodel is discussed as reflecting “chance”. Given that many links likely remain unobserved in ecological network datasets, a reasonable simulation of chance should ideally consider detection probability. Binary network data (e.g. the data used here) are often problematic: ecological network data are virtually always just samples of all realized interactions – this likely applies to the examples used here (even expert opinion may be influenced by observation bias). This can lead to strong biases in measures of network structure between the real web and its sample, but these problems are ignored here. As the simulations are called “pseudo-random”, they may be acceptable within the constraints of binary data – which then casts questions about the usefulness of the test dataset for the study purpose (see above).

**Unipartite vs. bipartite webs:**

It should be better explained how the different data types were handled with the same methods. Bipartite networks have additional (conceptual) ambiguities in how modularity should be calculated, which may be a core reason for discrepancy between modularity methods ( Guimera *et al*., 2007, Thébault, 2013). To be able to interpret the data better, it is warranted to present or identify the unipartite and bipartite webs separately in the graphs and results.

**Minor points:**

- More information on the datasets should be provided; the bipartite dataset is not even found in the reference provided for it, but must be traced back several steps to the original reference.
- Why is an algorithm chosen that is recommended for large networks (many thousands to millions of nodes, Blondel *et al.*, 2008) when the webs analyzed here have less than 200 nodes?
- Without defining the purpose or demonstrating the functional meaning of Q'R, it is difficult to know whether no correlation with connectance is desirable or not.
- “Results” should actually be entitled “Results and Discussion”.
- The terminological differentiation between true modularity and realized modularity is confusing.

Overall, the study is inconsistent and doesn’t live up to its promises. A study evaluating modularity measures should look at additional information to validate it (especially if it is not a formal comparison of multiple metrics). As shown by previous papers on the topic, this additional information could be the correspondence to biological traits in empirical networks (e.g. Martín González *et al.*, 2012), the detection of build-in module structure in simulated networks (e.g. Thébault, 2013) or the demonstration of functional consequences (e.g. by a model). To be useful, the study should be put on a more solid foundation.

I have read this submission. I believe that I have an appropriate level of expertise to confirm that it is of an acceptable scientific standard, however I have significant reservations, as outlined above.

# Peer review: Referee response for version 2

**DOI:** 10.5256/f1000research.2230.r1638

**Published:** 2013-09-02

**Authors:** Daniel Carstensen

**Affiliations:** 1 Department of Bioscience, Universidade Federal de São Paulo, São Paulo, Brazil

I have read this submission. I believe that I have an appropriate level of expertise to confirm that it is of an acceptable scientific standard.

# Peer review: Referee response for version 1

**DOI:** 10.5256/f1000research.1000.r1055

**Published:** 2013-07-11

**Authors:** Daniel Carstensen

**Affiliations:** 1 Department of Bioscience, Universidade Federal de São Paulo, São Paulo, Brazil

The aim of the author is interesting and relevant. I am intrigued by the development of a method to quickly evaluate different modularity measures, and an *a posteriori *method might well be a good solution. 

Overall the manuscript is generally well written. However, it is not clear to me how much is gained with this approach. Poisot only uses one method to detect the module configuration ( Louvain 2008) and one method to calculate the modularity ( Newman & Girvan 2004). It would be interesting to explore if the Q _R_ differs markedly when applied to the results of different methods. It would also be good to see what existing modularity measures do when optimizing modularity; do they minimize between module links? This is why Q _R_ measures and the strong correlation in Figure 2 is not surprising. What is more interesting about Figure 2 is that it shows that below a certain value of Q (~ 0,2 ?) it is not sensible to talk about modularity even if the empirical data is more modular than a random system. In such cases, the presented method seems useful to evaluate results. 

**Other minor revisions**

- An earlier reference could be used for the use of modularity in biogeographic networks instead of Cummings *et al.* 2010 (reference 6). Cummings *et al.* does not handle modularity. THe author should onsider citing Carstensen & Olesen 2009.
- In the 'Data and analysis' section the statement starting on line six in this paragraph needs a reference.
- Null model: What is meant by generality/successors and vulnerability/predecessors?

I have read this submission. I believe that I have an appropriate level of expertise to confirm that it is of an acceptable scientific standard, however I have significant reservations, as outlined above.

# Peer review: Referee response for version 1

**DOI:** 10.5256/f1000research.1000.r1028

**Published:** 2013-06-26

**Authors:** Carsten Dormann

**Affiliations:** 1 Biometry and Environmental System Analysis, University Freiburg, Freiburg, Germany

The proposed index of modularity is of striking simplicity - and thus likely to be prone to artifacts. In the opening paragraph, Poisot forgot to mention that random networks are also modular. Thus, a Q_R > 0 means, in itself, nothing, as Poisot rightly assumes when employing a null model.

The typically log-normal abundance of species in nature will introduce apparent structure into networks, even if the links simply reflect probabilistic interactions (i.e. any species interacts more with a common than a rare species). Thus, without a null model correcting for number of species, for their abundance and for the possibility of random networks also being modular, any index may report only spurious, artefactual results. Poisot uses a null model, but because his example data are binary networks (containing no information about the strength of a link), the best he (or anyone) can do is to use a null model based on degrees, which is only a very poor reflection of the actual abundance. Given that often more than a third of the species in a network are singletons, I believe that their contributions to modularity are overemphasized by any binary measure.

**Suggested Revisions:**

 1. Simulate networks (ideally weighted ones) and compare their Q_R values to quantitative null models. How much information does Q_R (and Q) actually contain? 

2. Comparison of Q_R not only with Q and connectance but also with other network metrics, such as linkage density or dependence asymmetry  (and particularly those with a more or less clear ecological interpretation, such as H2'). The question, again, is: what does Q_R provide in addition to current metrics?

3. Gain in ecological knowledge (which follows from 1. and 2.): If there is additional information, what does it mean? Which ecological features (specialization, number of functionally similar species, number of trophic level, number of habitats sampled etc.) contribute to Q_R? (For example along the lines of Pocock *et al.* 2012, who work on different types of sub networks put together into one large, or Clauset *et al*. 2008. Are these different sub networks identifiable as modules? If so, what does Q_R stand for?)

I have read this submission. I believe that I have an appropriate level of expertise to confirm that it is of an acceptable scientific standard, however I have significant reservations, as outlined above.
